# Risk factors associated with *Leishmania* exposure among dogs in a rural area of Ilha Solteira, SP, Brazil

**DOI:** 10.1590/0037-8682-0059-2020

**Published:** 2020-09-11

**Authors:** Julio Cesar Pereira Spada, Diogo Tiago da Silva, Maria Luana Alves, Nicolás Céspedes Cárdenas, Osvaldo Frederico Inlamea, Glaucia Amorim Faria, Andrea Gonçalves Ferreira, Helio Ricardo Silva, Trícia Maria Ferreira de Sousa Oliveira, Wilma Aparecida Starke Buzetti

**Affiliations:** 1 Universidade Estadual Paulista, Faculdade de Engenharia de Ilha Solteira, Departamento de Biologia e Zootecnia, Ilha Solteira, SP, Brasil.; 2 Universidade de São Paulo, Faculdade de Medicina Veterinária e Zootecnia, Programa de Pós-graduação em Epidemiologia Experimental Aplicada às Zoonoses, São Paulo, SP, Brasil.; 3 Universidade Estadual Paulista, Faculdade de Engenharia de Ilha Solteira, Departamento de Matemática, Ilha Solteira, SP, Brasil.; 4 Universidade de São Paulo, Faculdade de Zootecnia e Engenharia de Alimentos, Departamento de Medicina Veterinária, Pirassununga, SP, Brasil.

**Keywords:** Risk factors, Leishmania, Dogs, Rural area

## Abstract

**INTRODUCTION:**

We sought to determine risk factors (RFs) associated with the presence of antibodies against *Leishmania* in dogs from a rural area of Ilha Solteira, SP, Brazil.

**METHODS:**

Serum samples were collected from 250 dogs and tested using indirect enzyme-linked immunosorbent assay (ELISA) and indirect immunofluorescence antibody tests (IFATs). Data concerning dogs, their environment, and their owners’ knowledge of leishmaniasis were collected using a questionnaire. To determine RFs for contact with the parasite, univariate statistical analysis based on chi-squared and Fisher’s exact tests, followed by logistic regression, was used.

**RESULTS:**

It was found that 79/250 (31.6%) of the dogs were positive by IFAT, and 72/250 (28.8%) by ELISA. A total of 82/250 dogs (32.8%) were positive in at least one test. The RFs associated with occurrences of *Leishmania* exposure were large body size (OR = 2.25; 95% CI = 1.26-4.04; p = 0.003), presence of chickens (OR = 1.94; 95% CI = 1.05-3.65; p = 0.023), and lack of knowledge about *Leishmania* among dog owners (OR = 1.74; 95% CI = 0.96-3.21; p = 0.049). After multivariate analysis, the RFs for occurrence of *Leishmania* exposure in dogs that remained significantly associated were the dog’s size (large dogs) (OR = 1.2; 95% CI = 1.06-1.35; p = 0.003) and presence of chickens on the properties (small farms) (OR = 1.15; 95% CI = 1.02-1.30; p = 0.023).

**CONCLUSIONS:**

These results may be useful for improving preventive practices to reduce the incidence of *Leishmania* exposure among dogs in rural areas.

## INTRODUCTION

Leishmaniases are zoonoses caused by protozoa belonging to the genus *Leishmania* ^1^. The species that causes visceral leishmaniasis (VL) in countries in the Americas is *Leishmania infantum* (syn. *L. chagasi*)^2^. 

The main means of transmission of the parasite to dogs and other mammalian hosts is through the bite of females of hematophagous dipterans of the family Psychodidae belonging to the genera *Phlebotomus* and *Lutzomyia*, in the Old and New World, respectively, which are infected with promastigote forms of *Leishmania* spp.^3^ ^,^ ^4^. The species *Lutzomyia* (*Lutzomyia*) *longipalpis* is considered to be the main transmitter of the parasite in Brazil. This vector species feeds on a wide variety of vertebrate hosts, such as birds, wild and domestic mammals, and humans^5^.

Although several wild hosts have been identified in urban areas, *Canis familiaris* is the domestic host, and is considered to be the main reservoir of infection for humans^6^. Clinical manifestations of visceral canine leishmaniasis (CanL) are characterized by dermatological symptoms: flaking and excessive depigmentation, which normally develop on the head, and which differ to other parts of the body, with itching, dry skin, hair loss and areas of hyperkeratosis and lignification, and onychocryphosis. They may also present ulcers and small intradermal nodules, digestive symptoms (intestinal hemorrhage), respiratory symptoms (runny nose), eye symptoms (conjunctivitis, blepharitis, corneal enlargement and opacity) and general symptoms (apathy, anorexia, anemia, limb edema, hyporexia, weight loss and lymphadenomegaly)^7^. 

Many risk factors (RFs) for the occurrence of VL have been listed, thus indicating possible interactions between the links that make up the epidemiological chain, such as vectors, hosts and the environment^8^ ^,^ ^9^ ^,^ ^10^ ^,^ ^11^ ^,^ ^12^. Thus, knowledge of the distribution of the disease in endemic areas and possible associations between the disease and RFs can help in developing control strategies^13^. In this context, domestic dogs play an important role in the maintenance and spread of the disease. For this reason, factors that may be associated with the risk that these animals may become infected need to be well known^14^. 

Some studies conducted over the last decade have identified certain RFs that are associated with VL in urban regions. These include poor housing conditions, especially with a lack of household waste collection and an irregular or absent sewage system^15^; increased population density of phlebotomine sand-flies^15^ ^,^ ^16^; breeding of birds in cages in the presence of the vector^15^; and presence of other animals in the peridomestic area, particularly opossums^17^, chickens and pigs^18^.

Recently, a cross-sectional study carried out in endemic areas of Cuiabá, state of Mato Grosso, showed a CanL seroprevalence of 22.1%. Animals living in rural settings had a 1.9-fold higher risk of been infected than those in an urban environment. Factors relating to the habits of these animals, such as free access to the external environment and a watchdog function, along with the presence of agricultural activity were probably indicators that predicted *Leishmania* spp. exposure^19^.

Paulan et al. ^49^ (2012) used geoprocessing techniques in association with satellite imaging to reveal that the estimated prevalence of CanL in Ilha Solteira, state of São Paulo was low to medium-high, ranging from 10% to 14.5%, depending on the neighborhood studied. The areas with the highest density of CanL cases were close to natural vegetation fragments (at a zoo) and near rural settlements, i.e. farther from the city center. 

Spada et al. ^21^ (2014) studied the prevalence of *Lu. longipalpis* and CanL in the “Cinturão Verde” (green belt) area. They visited 12 properties over a 12-month period and collected biological samples from 32 dogs. Once a month, insects were caught using CDC (Centers for Disease Control and Prevention) traps. It was found that the vector was present on 100% of the properties, and that 31.25% of the dogs were positive for CanL.

The "Cinturão Verde" has a considerable human and canine population, which presents suitable conditions for vector maintenance, and is located near the urban perimeter of the city; this area represents an RF for maintenance of local disease and spread of this zoonosis to the urban area, if preventive measures are not implemented.

Thus, the objective of this study was to determine the RFs associated with *Leishmania* exposure among dogs in the “Cinturão Verde” of Ilha Solteira, SP, Brazil. 

## METHODS

### Study area

This study was conducted in a rural area referred to as the “Cinturão Verde” (Green Belt), which belongs to the municipality of Ilha Solteira (51°06’35” W and 20°38’44” S). The Cinturão Verde occupies an area of ​​880.46 hectares (ha) and is divided into agricultural production areas (563.29 ha); reforestation areas (317.68 ha); talvegues (lines connecting the lowest points of a river bed) (45.65 ha); area used for hydroelectric construction (227.39 ha); and legally enriched reserves (area with native vegetation cover) (44.12 ha). The entire extent of the Cinturão Verde is surrounded by 77 areas of dry land (non-irrigated) and 14 areas of irrigated land that are distributed among approximately 200 families. These families carry out various functional activities, such as growing vegetables and raising small animals, such as poultry and pigs. 

### Ethics Committee

The present study was approved by the Ethics Committee for Animal Use (CEUA) of the School of Engineering School of Ilha Solteira (part of São Paulo State University, UNESP). It formed part of a research project entitled "Distribution of the Phlebotomine Entomophase (Diptera: Psychodidae) and Canine Visceral Leishmaniasis Area of the “Cinturão Verde” of Ilha Solteira, State of São Paulo". Approval was granted at an ordinary meeting of CEUA held on May 9, 2011, under protocol no. 002/2011/CEUA. Procedures were performed based on current standards for research involving animal use according to the National Council for Animal Experiment Control (CONCEA).

### Study design and dog samples

A cross-sectional study on*Leishmania*exposure in dogs was conducted between February 2012 and February 2013. The sample size was established considering a population of 400 dogs (2 dogs/family) in the study area. Thus, the size of the sample, based on an arbitrary random method and with finite population adjustments of less than 200 dogs and a sampling error of 5%, was estimated to be approximately 250 dogs^22^. To ensure representativeness of the sample size, it was defined that the methodology for collecting the material should not involve any concentration of samples in any single region of the total area, but rather that the collection of material should cover the entire perimeter of the area. With the aid of a map provided by city authorities and a number of local visits to the study area, land areas and ownership were determined. In total, 104 families were visited, and biological material was collected from all dogs belonging to each family, irrespective of the numbers of dogs.

### Blood collection

Blood samples from the dogs were taken directly from the cephalic vein or the external jugular vein, using vacuum flasks without anticoagulant, to obtain serum samples. The whole blood was centrifuged at 900 × *g* for 10 minutes to separate the serum and was then kept at -20 °C until further use.

### Clinical Characterization

At the time of blood collection, the animals were examined clinically by means of general physical examination, and classified according to the clinical signs evident for CanL with one or more clinical signs and without clinical signs. Among the findings on physical examination, cachexia, hyperthermia, hyporexia, dermatological changes such as alopecia, ulcerative skin lesions, flaking, crusts, lymphadenomegaly, periocular lesions, uveitis, conjunctivitis, pale mucous membranes, and onychogryphosis were noted.

### Detection of anti-Leishmania antibodies

Anti-*Leishmania* antibodies were assayed using indirect immunofluorescence antibody tests (IFATs) and indirect enzyme-linked immunosorbent assays (ELISAs) as described by Oliveira et al. ^23^ (2008). Positive control serum was obtained from confirmed CanL cases that had been detected using direct methods. For negative controls, serum from healthy dogs was used. 

To perform the ELISA test, the soluble antigen of *L. infantum* was used at a concentration of 5 µg/mL, diluted in 0.05 M sodium carbonate bicarbonate buffer, pH 9.6. An anti-dog conjugate, rabbit anti-dog IgG coupled to alkaline phosphatase (Sigma Chemical Co, San Luis, Missouri, EUA) was diluted 1: 4000 in phosphate buffered saline (PBS), 0.01 M, pH 7.2 with 0.05% Tween-20 (PBS-Tween). As a substrate, paranitrophenylphosphate diluted to 1 mg/mL in diethanolamine buffer, pH 9.8 was used. The plates were read in an ELISA reader (Dynex Technologies, Chantilly, Virgínia, USA) at 405 nm. The cut-off point for the ELISA test corresponded to two and a half times the average value of the mean optical density (OD) of the negative reference sera. 

To perform IFATs, antigenic substrate was obtained from *L. infantum* promastigotes grown in RPMI - 1640 medium, at 25°C. Serial dilutions of each serum were performed commencing with a 1:40 dilution. The conjugate was dog anti-IgG linked to fluorescein isothiocyanate (KPL, Milford, *Massachusetts*, USA) diluted according to the manufacturer's recommendations. Sera were considered as positive when the parasites exhibited fluorescent color throughout the periphery, with a cut-off point of ≥ 1:40.

### Questionnaires

To determine RFs, standardized questionnaires were used. At the time of collection of blood from the dogs, the questionnaire was applied to the owners or caregivers of the animals. The information sought through the questionnaires included the identification and characteristics of the dogs, the environment in which the dogs lived, and the degree of knowledge regarding VL among the owners.

### Definition

Positivity for *Leishmania* exposure among the dogs was defined as positive detection of antibodies by means of IFAT or ELISA. 

### Geographic location of the animals

**FIGURE 1:**
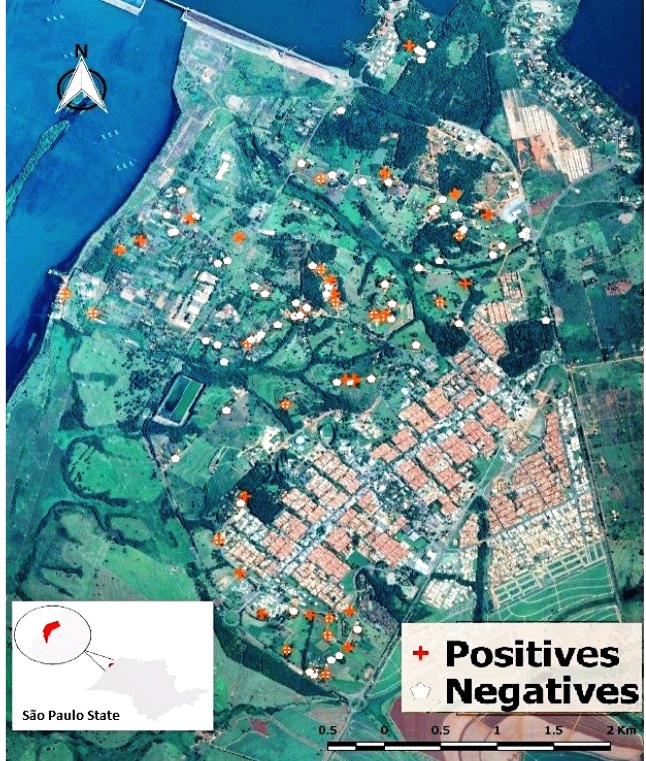
Geographic distribution of dogs in the rural area known as "Cinturão Verde" in the municipality of Ilha Solteira, state of São Paulo, Brazil, according to their serological condition. Serologically positive dogs are represented by red markings and negative dogs by white markings.

The locations were georeferenced using the Global Positioning System (GPS). These data were imported into a geographic information system (GIS) using the QGIS version 2.18.10 software package (Free Software Foundation, Boston, Massachusetts, EUA) with Open Layers plugin, to visualize the spatial distribution of the data. Finally, dots representing data points were projected into an image layer obtained from the Google Earth database (Figure 1).

### Statistical analysis

The association between potential RFs and *Leishmania* exposure in dogs was assessed by means of univariate analysis using the chi-squared test, or Fisher’s exact test, when required, and by means of multivariate logistic regression analysis. Odds ratios (OR) and 95% confidence intervals (CI) were calculated, and p values < 0.05 were considered statistically significant. All analyses were performed using R software (The R Foundation, Vienna, Austria), version 2.15.3^24^.

## RESULTS

A total of 250 dogs were analyzed. Anti-*Leishmania* antibodies were found in 79/250 (31.6%) of the dogs using IFAT, and 72/250 (28.8%) using ELISA. In total, 82/250 (32.8%) of the dogs were positive. 

According to clinical classifications, 192/250 (76.8%) dogs were classified as without clinical signs, and 58/250 (23.2%) as with clinical signs. Comparing the clinical conditions with the ratio of positive and negative dogs in the serological tests performed, it can be observed that although the high percentage of negative dogs in the group of dogs without clinical signs for both ELISA (150/192; 78.1%) and IFAT (146/192, 76.0%), there were positive dogs without clinical signs. Regarding dogs with clinical signs, some dogs in this group were negative for both tests performed. Positive dogs without clinical signs (21.9% and 23.9%) were less frequent than positive dogs with clinical signs (51.7% and 56.8%) for ELISA and IFAT, respectively (Table 1).

**TABLE 1:** Number (N) and percentage (%) of positive and negative dogs by serological methods (indirect ELISA and IFAT), according to classification as with and without clinical signs in the “Cinturão Verde” area, Ilha Solteira, SP, 2014.

Clinical Sign	With Clinical Signs (N = 58)		Without Clinical Signs (N = 192)		N = 250
	Positives	Negatives	Positives	Negatives	
Methods	N (%)	N (%)	N (%)	N (%)	N (%)
**ELISA**	30 (51,7)	28 (48,3)	42 (21,9)	150 (78,1)	72 (28,8)
**IFAT**	33 (56,9)	25 (43,1)	46 (23,9)	146 (70,1)	79 (31,6)

The ELISA cutoff point was an OD of 0.263 and, comparing the clinical classifications of the dogs with the average ELISA titers, it was found that positive dogs without clinical signs had an average OD of 0.642, and positive dogs with clinical signs had an average OD of 0.626. Regarding negative dogs, those with no manifestations exhibited an average OD of 0.254, and dogs with clinical signs had an average OD of 0.244.

Regarding the information obtained from the questionnaire, seven variables relating to dog characteristics were analyzed: hometown, use of repellent collar (impregnated with *4*% *deltamethrin)*, place where the dog slept (indoors or outdoors), age, size, sex and dog rearing (free or restrained). However, only animal size was significant by univariate analysis (Table 2) and in multivariate analysis (p < 0.05) (Table 3). Only large animals (such as German Shepherd, Rottweiler and similar breeds) were correlated with susceptibility to *Leishmania* exposure (OR = 2.25; 95% CI = 1.26-4.04; p = 0.003). Among the 82 samples that were positive for antibodies against *Leishmania*, 39 (44.8%) were in the group of large dogs. 

**TABLE 2:** Univariate association analysis of variables relating to risk factors associated with ***Leishmania* exposure**, based on dogs (N = 250) in the “Cinturão Verde” area. Ilha Solteira, SP, 2014.

Dog variables	Category	Positive	Negative	Proportion	OR	95% CI	P-value
**Municipality of origin**	Ilha Solteira	71	138	(0.836)	1.40	0.63-3.28	0.3731
	Other	11	30	(0.164)	1.0		
**Use of repellent collar**	No	80	166	(0.984)	0.48	0.03-6.78	0.5993
	Yes	2	2	(0.016)	1.0		
**Location where the dog sleeps**	Inside home	80	2	(0.328)	0.48	0.06-3.48	0.46
	In peridomestic area	2	166	(0.672)	1.0		
**Age**	< 6 months to 1 year	13	37	(0.2)	0.66	0.33-1.33	0.25
	Adult	69	131	(0.8)	1.0		
**Dog size**	Large	39	48	(0.38)	2.25	1.26-4.04	0.003*
	Small-medium	43	120	(0.652)	1.0		
**Habit**	Loose	45	74	(0.476)	1.54	0.87-2.74	0.1074
	Restrained	37	**94**	(0.524)	1.0		
**Gender**	Male	54	91	(0.58)	1.63	0.94-2.82	0.078
	Female	28	77	(0.42)	1.0		

**Note:** *chi-square test or Fisher’s exact test (significance p ≤ 0.05). OR = odds ratio; 95% CI = 95% confidence interval.

**TABLE 3:** Results of multivariate analysis on risk factors relating to seropositivity of dogs for *Leishmania* infection in the “Cinturão Verde” area. Ilha Solteira, SP, 2014.

Variable	Coefficient	Standard error	T-value	OR	95% CI	P-value*
**Dog size**	0.18201	0.06098	2.985	1.20	1.06-1.35	0.003
Presence of chickens	0.13744	0.06023	2.282	1.15	1.02-1.30	0.023

**Note:** *The following variables were included in the multivariate analysis model: dog size. presence of chickens, and lack of knowledge regarding CVL among owners.

Among the 250 dogs sampled in this study, there were 145 males and 105 females. There was greater occurrence of *Leishmania* exposure in the male population (54/82; 65.9%), but this difference was not significant (OR = 1.63; 95% CI = 0.94-2.82; p = 0.079). 

Regarding the age of the animals, 200 (80.0%) were adults (more than one year of age), while 50 (20.0%) were less than one year old. Although the positivity rate was higher among adult animals (69/82; 84.2%), i.e. those over one year of age, the difference was not statistically significant (OR = 0.66; 95% CI = 0.33-1.33; p = 0.250). The small number of young dogs observed in this study suggests that there was little rotation or replacement of dogs in the area studied. 

Regarding the habits of these animals, there was no significance (OR = 2.07; 95% CI = 0.15-2.64; p = 0.599). However, it was observed that during the night, among the 82 positive animals, two dogs slept indoors and 50 outside in the yard, and also that among these 82 positive dogs, 37 were restrained and 45 were loose on the properties. 

Regarding the environment (Table 4), of the 82 animals that were positive for *Leishmania* exposure, all lived with other animals (60 with poultry, 29 with cats, 27 with pigs, 24 with horses and 13 with cattle). In the present study, the presence of poultry (hens) cohabiting with dogs was shown to be another RF that influenced the presence or maintenance of infected dogs in the rural regions, as shown by univariate (Table 4) and multivariate analyses (Table 3) (OR = 1.94; 95% CI = 1.05-3.65; p = 0.023). It was observed that of the 82 seropositive dogs, 60 lived on properties on which chickens were also kept. 

**TABLE 4:** Univariate association analysis of variables relating to risk factors associated with ***Leishmania* exposure**, based on environmental factors (N = 250 dogs) in the “Cinturão Verde” area. Ilha Solteira, SP, 2014.

Variables	Category	Positive	Negative	Proportion	OR	95% CI	P-value
**Report of dog euthanized due to the disease**	No	41	106	(0.74)	0.53	0.26-1.1	0.05
	Yes	22	30	(0.26)	1.0		
**Presence of cats**	Yes	29	51	(0.32)	1.25	0.68-2.27	0.4254
	No	53	117	(0.68)	1.0		
**Presence of cattle**	Yes	13	21	(0.13)	1.31	0.57-2.94	0.46
	No	69	147	(0.86)	1.0		
**Presence of horses**	Yes	24	36	(0.24)	1.51	0.78-2.87	0.17
	No	58	132	(0.76)	1.0		
**Presence of pigs**	Yes	27	56	(0.33)	0.98	0.53-1.77	0.94
	No	55	112	(0.67)	1.0		
**Presence of chickens**	Yes	60	98	(0.63)	1.94	1.05-3.65	0.02238*
	No	22	70	(0.37)	1.0		
**Garbage collection**	No	20	37	(0.23)	1.14	0.57-2.21	0.6754
	Yes	62	131	(0.78)	1.0		
**Presence of forest**	Yes	65	132	(0.79)	1.04	0.52-2.13	0.8993
	No	17	36	(0.21)	1.0		
**Diverse vegetation**	Yes	73	153	(0.90)	0.79	0.30-2.16	0.6059
	No	9	15	(0.09)	1.0		
**Real estate management**	Yes	16	35	(0.20)	0.92	0.44-1.85	0.8077
	No	66	133	(0.80)	1.0		
**Growing of tubers**	Yes	18	38	(0.22)	0.96	0.47-1.88	0.9054
	No	64	130	(0.78)	1.0		
**Fruit growing**	Yes	20	37	(0.23)	1.14	0.57-2.21	0.6754
	No	62	131	(0.78)	1.0		
**Cultivation of vegetables**	No	7	27	(0.14)	0.48	0.17-1.22	0.1027
	Yes	75	141	0.864	1.0		
**Accumulation of organic matter**	Yes	74	160	(0.94)	0.46	0.14-1.47	0.1298
	No	8	8	(0.06)	1.0		

**Note:** *chi-square test or Fisher’s exact test (significance p ≤ 0.05). OR = odds ratio; 95% CI = 95% confidence interval.

It was found that 20.8% of the dogs sampled were on properties that reported dogs having been put down because of *Leishmania* exposure (OR = 0.53; 95% CI = 0.26-1.10; p = 0.055). This indicated that the disease was present in this rural environment and that greater attention to it needs to be paid by health authorities. None of the properties reported that any of their dogs had been treated for VL. Among the 82 seropositive dogs, 20 lived on properties that did not have any selective garbage collection. 

Finally, regarding the owners’ knowledge concerning VL (Table 5), it was found that 27.2% of the dogs were under the ownership of people who did not know about the disease and how it is transmitted (72.8%); or about its severity and lethality, not only in relation to dogs but also to humans (60.8%). This lack of knowledge concerning the disease among rural populations was a RF for the disease by univariate analyses (OR = 1.74; 95% CI = 0.96-3.21; p = 0.049), whereas this factor ceased to be significant after multivariate analysis. However, it should be noted here that a large proportion (72.8%) of the rural population interviewed reported having a lack of knowledge about the role of the insect vector in relation to transmission of leishmaniasis, and regarding dogs being the main domestic reservoir (64.4%). In addition, 49.2% of the interviewees answered that they were totally unaware of the presence of the vector on their properties, thus corroborating the hypothesis that knowledge concerning CanL among the human population is a crucial factor regarding its prevention. 

**TABLE 5:** Univariate association analysis of variables relating to risk factors associated with seropositivity of dogs for ***Leishmania* exposure** and with their owners’ knowledge about the disease (N = 250 dogs) in the “Cinturão Verde” area. Ilha Solteira, SP, 2014.

Variables	Category	Positive	Negative	Proportion	OR	95% CI	P-value
**Ever heard of the disease?**	No	23	45	(0.27)	1.06	0.56-1.99	0.8331
	Yes	59	123	(0.728)	1.0		
**Do you know how it is acquired?**	No	61	121	(0.73)	1.13	0.59-2.18	0.6930
	Yes	21	47	(0.27)	1.0		
**Do you know that it can attack humans?**	No	57	95	(0.61)	1.74	0.96-3.21	0.04869*
	Yes	25	73	(0.40)	1.0		
**Do you know about the role of dogs in its transmission?**	No	36	119	(0.62)	1.36	0.772-2.67	0.3172
	Yes	19	49	(0.27)	1.0		
**Do you know about the role of the vector?**	No	59	102	(0.64)	1.67	0.90-3.09	0.08149
	Yes	23	66	(0.37)	1.0		

**Note:** *chi-square test or Fisher’s exact test (significance p ≤ 0.05). **OR:** odds ratio; 95%, **CI:** 95% confidence interval.

## DISCUSSION

In other studies carried out in Ilha Solteira, the prevalence of CanL in urban areas ranged from 10% to 14%^20^ ^,^ ^25^, while it was 37.7% rurally ^26^ and 89% at animal shelters of the Association for Animal Protection^27^.

Here, there were seropositive dogs with and without clinical signs for both serological tests performed, but with a greater number of positive dogs with clinical manifestations (51.7% and 56.8%) than positive dogs without clinical signs: 21.9% and 23.9% for ELISA and IFAT, respectively. According to other authors, a large majority of dogs without clinical signs are negative in the different tests routinely used for CanL detection^28^ ^,^ ^29^ ^,^ ^30^. However, dogs without clinical signs but positive for CanL may be infectious for sand-flies in a proportion similar to those with clinical signs, and are equally important in the epidemiological chain of the disease^31^ ^,^ ^32^. 

Some studies have shown that dogs without clinical signs are generally seronegative, or have antibody levels that are difficult to detect in serological tests. On the other hand, dogs with clinical signs generally exhibit high levels of antibodies^33^. In the present study, antibodies against *Leishmania* were detect by ELISA, with a similar mean OD between groups of dogs with and without clinical signs. Portela et al^34^. (2019) highlighted the importance of adequate diagnosis of infected dogs without clinical signs in endemic areas, since these dogs can remain untreated or unattended. Laranjeira et al. ^35^ (2014) point out that infected dogs lacking clinical signs can develop active infections, representing a source of infection for other dogs and humans, and that even after a recent infection, they produce specific antibodies at high levels before developing clinical signs. Likewise, Assis et al. ^36^ (2010) identified an animal without clinical signs and showing high titrates on ELISA and IFAT tests, with moderate to intense parasitic grades in the spleen and liver tissues, respectively, by immunohistochemical and histochemical examination.

During an epidemiological evaluation of the southeastern and southern regions of Spain, it was observed that the seroprevalence of CanL gradually increases with the size of the animal. This characteristic was found to be an RF for the disease^37^, which supports our results. Besides the fact that all dogs are susceptible to *Leishmania* exposure, Feitosa et. al. ^7^ (2000) also correlated greater frequency of CanL cases with larger dogs. Since these serve as guard dogs, they are kept in peridomestic areas, and thus are probably more exposed to the vector. Penaforte et al. ^38^ (2013) also studied this association and suggested that large dogs suffer more sand-fly bites because they are used as guard dogs, living outside houses. 

Almeida (2012)^12^ and Figueiredo et. al. ^39^ (2014) also found no gender-based predisposition. However, Medeiros et al. ^40^ (2008) observed greater predisposition among male dogs, while Amóra et al. (2006) found a higher percentage of *Leishmania* infections among bitches in rural areas.

Contrary to our research, Figueiredo et al. ^39^ (2014) found higher positivity among young dogs. Similarly, Moreno; Alvar^42^ (2002) found that dogs younger than three years of age and older than seven were at higher risk of contracting CanL, and that the first of these groups was more susceptible than the second.

Amorá et al.^41^ (2006) observed that the greatest number of seropositive dogs had semi-domestic habits, which they explained by suggesting that these animals are more exposed to vector action. Some research has shown that dogs from endemic areas exposed at night can be stung by hundreds of sand-flies. This continuous exposure may favor seroconversion and development of the disease, since the parasite is continuously introduced into the skin of these animals^43^.

Although other vertebrate animals can serve as food sources for sand-flies, and favor their maintenance in areas close to homes, some, such as chickens, can also reduce the number of infectious bites in dogs^44^. However, Azevedo et al. ^45^ (2008), verified that association of the prevalence of seropositivity in dogs is related to cohabitation with other species: chickens were the most frequent cohabitees among the positive dogs, followed by pigs and horses. Borges et al. ^46^ (2009) demonstrated that poultry have immense potential for attracting sand-flies, and noted that chickens deserve special attention because of their higher frequency among households, as well as their potential for generating a favorable environment for procreation of sand-flies because of the organic waste that they produce. In another study, Barboza et al. ^47^ (2009) observed the co-presence of chickens, pigs and horses, but found that cats were the most frequent cohabitees with seropositive dogs. 

Recently, a cross-sectional study conducted in endemic areas of Cuiabá (MT) has shown that dogs living in rural settings were 1.9 times more likely to acquire the infection than were those in urban environments^19^. Factors relating to the dogs’ habits, such as free access to the streets and serving a guard function, as well as the presence of agricultural activity, were considered to be indicators that predicted infection by *Leishmania* spp. In addition, Costa et al. ^48^ (2005) and Moreno et al. ^15^ (2005) observed that poor housing conditions, open sewage ditches, lack of household waste collection, and irregular or absent disposal of sewage were RFs for *Leishmania* infection in urban areas.

Moreno et al. ^15^ (2005) reported in a study conducted in the metropolitan region of Belo Horizonte that the likelihood that a population would be affected by CanL was six times higher for people who did not know about the vector than for those who were aware of it.

Our data have reinforced the hypothesis that many people still have poor knowledge regarding leishmaniasis and how it is transmitted. This corroborates a recent report by Paulan et al. ^49^ (2016), who found that rural families established in the "Estrela da Ilha" rural settlement in Ilha Solteira, SP, presented fragmented knowledge concerning the disease, thus resulting in inefficient practices of prophylactic measures against leishmaniasis among humans and dogs in this rural area.

Changes in attitudes in populations is a goal to be achieved over time, since this involves cultural changes, which seem to be a crucial factor regarding the difficulty in attaining control over this zoonosis. According to Borges et al. ^50^ (2008), knowledge of the forms of VL transmission and vector recognition decreases the risk of contracting leishmaniasis by a factor of 0.79, while lack of knowledge about the disease increases the risk by a factor of 2.57.

## CONCLUSIONS

The RFs associated with occurrence of *Leishmania* exposure in domestic dogs on properties of the “Cinturão Verde” in Ilha Solteira, SP, were large body size among the dogs, the presence of chickens, and lack of knowledge regarding *Leishmania* among dog owners. After adjustment through multivariate analysis, only dog size and the presence of chickens were related to the presence of *Leishmania* exposure among the dogs. However, we must emphasize that this probably happened due to the fact that most of the local population had no knowledge about the disease.

## References

[1] Ross R (1903). (1) note on the bodies recently described by Leishman-Donovan and
(2) Further notes on Leishman’s bodies. Br Med J.

[2] Kuhls K, Alam MZ, Cupolillo E, Ferreira GEM, Mauricio IL, Oddone R (2011). Comparative microsatellite of new world Leishmania infantum
reveals low heterogeneity among populations and recent old world
origin. PLoS Negl Trop Dis.

[3] Deane LM, Deane MP (1955). Leishmaniose visceral urbana (no cão e no homem) em Sobral,
Ceará. O Hospital.

[4] Young DG, Duncan MA (1994). Guide to the identification and geographic distribution of Lutzomyia
sand flies in Mexico, the West Indies, Central and South America (Diptera:
Psychodidae).

[5] Ministério da Saúde (MS). Secretaria de Vigilância em Saúde.
Departamento de Vigilância Epidemiológica (2014). Manual de Vigilância e Controle da Leishmaniose Visceral.

[6] Gontijo CMF, Melo MN (2004). Leishmaniose visceral no Brasil: quadro atual, desafios e
perspectivas. Rev Bras Epidemiol.

[7] Feitosa MM, Ikeda FA, Luvizotto MCR, Perri SHV (2000). Aspectos clínicos de cães com leishmaniose visceral no município
de Araçatuba, São Paulo, Brasil. Clín Vet.

[8] Dye C (1992). Leishmaniasis epidemiology: the theory catches up. Parasitol.

[9] Gavgani ASM, Mohite H, Edrissian GH, Mohebali M, Davies CR (2002). Domestic dog ownership in Iran is a risk factor for human
infection with Leishmania Infantum. Am J Trop Med Hyg.

[10] Monteiro EM, Silva JCF, Costa RT, Costa DC, Barata RA, Paula EV (2005). Leishmaniose visceral: estudos de flebotomíneos e infecção canina
em Montes Claros, Minas Gerais. Rev Soc Bras Med Trop.

[11] Murray HW (2005). Advances in leishmaniasis. Lancet.

[12] Rondon FCM, Bevilaqua CML, Franke CR, Barros RS, Oliveira FR, Alcântara AC (2008). Cross-sectional serological study of canine Leishmania infection
in Fortaleza, Ceará state, Brazil. Vet Parasitol.

[13] Frehse MS, Greca H, Ullmann LS, Camossi LG, Machado JG, Langoni H (2010). Surveillance of canine visceral leishmaniasis in a disease-free
area. Rev Bras Parasitol Vet.

[14] Dantas TF (2009). Canine leishmaniosis in South America. Parasit Vectors.

[15] Moreno EC, Melo MN, Genaro O, Lambertucci JR, Serufo JC, Andrade ASR (2005). Risk factors for Leishmania chagasi infection in an urban área of
Minas Gerais State. Rev Soc Bras Med Trop.

[16] França-Silva JC, Barata RA, Costa RT, Monteiro EM, Machado-Coelho GLL, Vieira EP (2005). Importance of Lutzomyia longipalpis in the dynamics of
transmission of canine visceral leishmaniasis in the endemic area of
Porteirinha municipality, Minas Gerais, Brazil. Vet Parasitol.

[17] Cabrera MA, Paula AA, Camacho LAB, Marzochi MCA, Xavier SC, Silva AVM (2003). Canine visceral leishmaniasis in Barra do Guaratiba, Rio de
Janeiro, Brazil: assessment of risk factors. Rev Inst Med Trop S Paulo.

[18] Moreira ED, Souza VMM, Sreenivasan M, Lopes N, Barreto RB, Carvalho LP (2003). Peridomestic risks factors for canine leishmaniasis in urban
dwellings: new findings from a prospective study in Brazil. Am J Trop Med Hyg.

[19] Almeida ABPF, Sousa VRF, Cruz FACS, Dahroug MAA, Figueiredo FB, Madeira MF (2012). Canine visceral leishmaniasis: seroprevalence and risk factors in
Cuiabá, Mato Grosso, Brazil. Rev Bras Parasitol Vet.

[20] Paulan SC, Silva HR, Lima EAF, Flores EF, Tachibana VM, Kana C (2012). Spatial distribution of Canine Visceral Leishmaniasis in Ilha
Solteira, São Paulo Brazil. Eng Agríc.

[21] Spada JCP, Silva DT, Martins KRR, Rodas LAC, Alves ML, Faria GA (2014). Occurrence of Lutzomyia longipalpis (Phlebotominae) and canine
visceral leishmaniasis in a rural area of Ilha Solteira, SP,
Brazil. Rev Bras Parasitol Vet.

[22] Kish L (1965). Survey Sampling.

[23] Oliveira TMFS, Furuta PI, de Carvalho D, Machado RZ (2008). A study of cross-reactivity in serum samples from dogs positive
for Leishmania sp., Babesia canis and Ehrlichia canis in enzyme-linked
immunosorbent assay and indirect fluorescent antibody test. Rev Bras Parasitol Vet.

[24] R Core Team (2017). R: a language and enviroment for statistical computing.

[25] Pereira VF, Benassi JC, Starke-Buzetti WA., Silva DT, Ferreira HL, Keid LB (2016). Detection of canine visceral leishmaniasis by conjunctival swab
PCR. Rev Soc Bras Med Trop.

[26] Paulan SC, Lins AGS, Tenório MS, Pena HFJ, Machado RZ, Gennari SM (2013). Seroprevalence rates of antibodies against Leishmania infantum
and other protozoan and rickettsial parasites in dogs. Rev Bras Parasit Vet.

[27] Silva DT, Starke-Buzetti WA, Alves-Martin MF, Paixão MS, Tenório MS, Lopes MLM (2014). Comparative evaluation of several methods for Canine Visceral
Leishmaniasis diagnosis. Rev Bras Parasitol Vet.

[28] Assis J, Queiroz NMGP, Silveira RCV, Nunes CM, Oliveira TMFS, Noronha-Junior ACF (2010). Estudo comparativo dos métodos diagnósticos para leishmaniose
visceral em cães oriundos de Ilha Solteira, SP. Rev Bras Parasit Vet.

[29] Queiroz NMGP, Assis J, Oliveira TMFS, Machado RZ, Nunes CM, Satrke-Buzeti WA (2010). Diagnóstico da leishmaniose visceral canina pelas técnicas de
imunoistoquímica e PCR em tecidos cutâneos em associação com a RIFI e
ELISA-teste. Rev Bras Parasitol Vet.

[30] Quinnell RJ, Carson C, Reithinger R, Garcez LM, Courtenay O (2013). Evaluation of rK39 rapid diagnostic tests for canine visceral
leishmaniasis: longitudinal study and meta-analysis. PLoS Negl Trop Dis.

[31] Laurenti MD, Rossi CN, da Matta VL, Tomokane TY, Corbett CE, Secundino NF (2013). Asymptomatic dogs are highly competent to transmit Leishmania
(Leishmania) infantum chagasi to the natural vector. Vet Parasitol.

[32] Manna L, Reale S, Viola E, Vitale F, Manzillo VF, Michele PL (2006). Leishmania DNA load and cytokine expression levels in
asymptomatic naturally infected dogs. Vet Parasitol.

[33] Solano-Gallego L, Koutinas A, Miró G, Cardoso L, Pennisi MG, Ferrer L (2009). Directions for the diagnosis, clinical staging, treatment and
prevention of canine leishmaniosis. Vet Parasitol.

[34] Portela RWD, Soares RP, Passos GP, Laranjeira DF, Barral TD, Sampaio JR (2019). Leishmania infantum-derived lipophosphoglycan as an antigen in
the accurate serodiagnosis of canine leishmaniasis. PLoS Negl Trop Dis.

[35] Laranjeira DF, Da Matta VLR, Tomokane TY, Marcondes M, Corbet CEP, Laurenti MD (2014). Serological and infection statuses of dogs from a visceral
leishmaniasis-endemic area. Rev. Saude Publ.

[36] Assis J, Queiroz NMP, Silveira RCV, Nunes CM, Oliveira TMFS, Noronha J (2010). Estudo comparativo dos métodos diagnósticos para Leishmaniose
Visceral em cães oriundos de Ilha Solteira, SP. Rev Bras Parasitol Vet.

[37] Galvez R, Miro G, Descalzo MA, Nieto J, Dado D, Martin O (2010). Emerging trends in the seroprevalence of canine leishmaniasis in
the Madrid region (central Spain). Vet Parasitol.

[38] Penaforte KM, Belo VS, Teixeira-Neto RG, Ribeiro RAN, Oliviera RB, Schettini DA (2013). Leishmania infection in a population of dogs: an epidemiological
investigation relating to visceral leishmaniasis control. Rev Bras Parasitol Vet.

[39] Figueiredo MJFM, Souza NF, Figueiredo HF, Meneses AMC, Silva-Filho E, Nascimento GG (2014). Fatores de risco e classificação clínica associados à
soropositividade para leishmaniose visceral canina. Rev Bras Saúde Prod Anim.

[40] Medeiros CFO, Melo AGC, Lima AKF, Silva ING, Oliveira LC, Silva MC (2008). Perfil hematológico de cães com leishmaniose visceral no
município de Fortaleza, Ceará. Ciênc Anim.

[41] Amóra SSA, Santos MJP, Alves ND, Costa SCG, Calabrese KS, Monteiro AJ (2006). Fatores relacionados com a positividade de cães para leishmaniose
visceral em área endêmica do Estado do Rio Grande do Norte,
Brasil. Ciênc Rural.

[42] Moreno J, Alvar J (2002). Canine leishmaniasis: epidemiological risk and the experimental
model. Trends Parasitol.

[43] Solano-Gallego L, Koutinas A, Miro G, Cardoso L, Pennisi MG, Ferrer L (2009). Directions for the diagnosis, clinical staging, treatment and
prevention of canine leishmaniosis. Vet Parasitol.

[44] Silva RBS, Porto ML, Barbosa WO, Souza HC, Marques NFSP, Azevedo SS (2018). Seroprevalence and risk factors associated with canine visceral
leishmaniasis in the State of Paraíba, Brazil. Rev Soc Bras Med Trop.

[45] Azevedo MAA, Dias AKK, Paula HB, Perri SHV, Nunes CM (2008). Avaliação da leishmaniose visceral canina em Poxoréo, Estado do
Mato Grosso, Brasil. Rev Bras Parasitol Vet.

[46] Borges BKA, Silva JAS, Haddad JPA, Moreira EC, Magalhãaes DF, Ribeiro LML (2009). Presença de animais associada ao risco de transmissão da
leishmaniose visceral em humanos em Belo Horizonte, Minas
Gerais. Arq Bras Med Vet Zootec.

[47] Barboza DCPM, Leal DC, Souza BMPS, Carneiro AJB, Gomes CMB, Alcânatara ACD (2009). Inquérito epidemiológico da leishmaniose visceral canina em três
distritos sanitários do Município de Salvador, Bahia, Brasil. Rev Bras Saúde Prod Anim.

[48] Costa CHN, Werneck GL, Jr. L Rodrigues, Santos MV, Araújo IB, Moura LS (2005). Household structure and urban services: neglected targets in the
control of visceral leishmaniasis. Ann Trop Med Parasitol.

[49] Paulan SC, Silva DT, Lins AGS, Lima FL, Tenório MS, Tasca KI (2016). O conhecimento sobre leishmaniose visceral: suficiente para
controle e prevenção?. Rev Ciênc Ext.

[50] Borges BKA, Silva JAS, Haddad JPA, Moreira EC, Magalhãaes DF, Ribeiro LML (2008). Avaliação do nível de conhecimento e de atitudes preventivas da
população sobre a leishmaniose visceral em Belo Horizonte, Minas Gerais,
Brasil. Cad de Saúde Pública.

